# The ABCC6 transporter: what lessons can be learnt from other ATP-binding cassette transporters?

**DOI:** 10.3389/fgene.2013.00203

**Published:** 2013-10-16

**Authors:** Olivier M. Vanakker, Mohammad J. Hosen, Anne De Paepe

**Affiliations:** Center for Medical Genetics, Ghent University HospitalGhent, Belgium

**Keywords:** pseudoxanthoma elasticum, ABCC6, ABC transporters, substrate identification, clinical variability, integrated approach, systems biology, modifier genes

## Abstract

ABC transporters represent a large family of ATP-driven transmembrane transporters involved in uni- or bidirectional transfer of a large variety of substrates. Divided in seven families, they represent 48 transporter proteins, several of which have been associated with human disease. Among the latter is ABCC6, a unidirectional exporter protein primarily expressed in liver and kidney. ABCC6 deficiency has been shown to cause the ectopic mineralization disorder pseudoxanthoma elasticum (PXE), characterized by calcification and fragmentation of elastic fibers, resulting in oculocutaneous and cardiovascular symptoms. Unique in the group of connective tissue disorders, the pathophysiological relation between the ABCC6 transporter and ectopic mineralization in PXE remains enigmatic, not in the least because of lack of knowledge on the substrate(s) of ABCC6 and its unusual expression pattern. Because many features, including structure and transport mechanism, are shared by many ABC transporters, it is worthwhile to evaluate if and to what extent the knowledge on the physiology and pathophysiology of these other transporters may provide useful clues toward understanding the (patho)physiological role of ABCC6 and how its deficiency may be dealt with.

In this paper, we will summarize relevant knowledge and methods for analysis on ABC transporters which may be useful for the further study of ABCC6.

Pseudoxanthoma elasticum is an autosomal recessive disorder resulting from aberrant mineralization and fragmentation of elastic fibers in the extracellular matrix of the skin, the eyes and the cardiovascular system. It is characterized by papular skin lesions, a retinopathy prone to hemorrhage due to subretinal neovascularisation, and premature occlusive vessel disease, with considerable inter- and intra-familial variability of severity (Vanakker et al., 2008). Thirteen years after the discovery of the causal relation between *ABCC6* mutations and PXE, significant progress has been made in the characterization of the *ABCC6* gene and the transporter it encodes (Bergen et al., 2000; Le Saux et al., 2000). Nevertheless, ABCC6 remains surrounded by a number of unsolved enigmas, including its substrates and physiological functions as well as the mechanisms by which it is connected to ectopic elastic fiber mineralization. Further, the significant variability in clinical severity of the PXE phenotype cannot be explained by the *ABCC6* mutations, as demonstrated by numerous negative genotype-phenotype correlation studies (Meloni et al., 2001; Gheduzzi et al., 2004; Chassaing et al., 2005; Pfendner et al., 2007). This suggested the involvement of modifier genes, influencing particularly the severity of PXE symptoms (Zarbock et al., 2009).

Despite marked structural resemblance with other ABC transporters, ABCC6 has been considered the odd man out, due to its unique relation with a connective tissue disease affecting tissues in which it is hardly expressed. Drawing analogies between ABCC6 and the other ABC transporters has been done in the past, particularly to gain insights in substrate interactions. While, after more than a decade, the identification of this substrate is being further pursued, it may be a good time to evaluate if information on other ABC transporters as well as the difficulties that were and are still encountered in their study may point out potential pitfalls, concepts and approaches on how to deal with the challenges in the field of PXE.

## THE PHYSIOLOGICAL FUNCTIONS OF ABCC6

Perhaps one of the most intriguing questions surrounding ABCC6 is the nature of its substrate and hence the physiological function of the transporter. Following unsuccessful comparisons, it is doubtful that the function of other members of the ABC transporter family will imply anything concerning the actual function of ABCC6 (Madon et al., 2000). It has been previously demonstrated in plant ABC transporters that – despite phylogenetic similarities and domain homologies – related ABC transporters can in fact serve diverse physiological functions. In humans, this was endorsed through the comparison of the cystic fibrosis transmembrane conductance regulator (ABCC7 or CFTR, the gene for cystic fibrosis) and P-glycoprotein (ABCB1 or MDR – multidrug resistance protein; Luckie et al., 2003). This however does not imply that nothing can be learned from previous experiences with functional dissection of ABC transporters. Awareness has risen for several of these that the complexity of (patho)physiological mechanisms related to the wild type and mutant transporter is much higher than anticipated; in this respect and based on our current knowledge, ABCC6 will probably not be an exception to the rule. This enlarging complexity began with the changing concept of substrate specificity. For several ABC transporters either one or a small number of closely related substrates are known. However, there have been reports of other transporters, such as ABCC1, ABCC2 or ABCB1, for which multispecificity for a diverse range of substrates has been shown (Jemnitz et al., 2010; He et al., 2011). The diversity of (patho)physiological observations in PXE – oxidative stress, the PXE serum factor, vitamin K deficiency, the relation with *ENPP1*, does not make it unthinkable that they result from aberrant transport of more then one substrate, thus influencing more than one physiological process (Le Saux et al., 2006; Pasquali-Ronchetti et al., 2006; Vanakker et al., 2010; Nitschke et al., 2011). For many ABC transporters – including ABCC6 – the specific sequences responsible for substrate recognition have not yet been identified, which may add to the complexity of substrate identification (Glavinas et al., 2004). Nevertheless, for some transporters such as ABCC2 and Pgp, binding-site models have been proposed usingrelatively high accuracy *in silico* methods. Such models include the presence of one large binding site and multiple smaller ones, primary and secondary binding sites or three distinct sites (Hirono et al., 2005; Borst et al., 2006; Pedersen et al., 2008; Ferreira et al., 2013).

These *in silico* approaches such as molecular docking, though insufficiently reliable to pinpoint with certainty the physiological substrates, may also have the advantage to generate a list of potential substrates in a cost- and time-efficient manner. Subsequently, these predictions need to be validated *in vivo*. Several approaches can be applied to identify physiological compounds transported by ABC proteins, such as *in vivo* hepatobiliairy elimination studies in mutant animal models, and using membrane vesicles or functional assays based on mass spectrometry (Ci et al., 2007; Katona et al., 2009; Jemnitz et al., 2010). Though murine model organisms are commonly used for such experiments and the *Abcc6 *knock-out mouse model largely recapitulates the clinical features of PXE, there have been reports emphasizing the differences in the physiological mechanisms between rodents and humans, which may slow down substrate identification (Ivan et al., 2013). This has been the case for the ABCG2 protein, recently discovered to be an urate transporter (Nakayama et al., 2011). It should remind us to be cautious to extrapolate findings (positive or negative) in other species to the human disease. Remembering the adage that humans remain the most optimal model to search for substrate(s), an interesting approach for substrate screening has been reported for ABCC2 (Krumpochova et al., 2012). To determine the extent of its substrate spectrum, a variant on the classic vesicular transport experiments has been applied to extract substrates from body fluids. Classic vesicular transport studies have as a drawback that an upfront hypothesis about the transported compounds is necessary and that only one substrate can be evaluated at a time. Certainly, the necessity of an upfront hypothesis is a major disadvantage with respect to ABCC6. By incubating the vesicles in body fluids (in the case of *Abcc2* murine urine) and analyzing the vesicle content by LC/MS, several novel compounds were identified. To apply this technique for ABCC6 would imply the use of human plasma and/or hepatocytes. This transportomics technique has great potential with several advantages including a reduced number of experimental animals and would mean that identification of compounds is not a prerequisite to study their transport in vesicular transport experiments as one can fish for new substrates. Combining transportomics with untargeted metabolomics analysis would further increasethe range of potential substrates that can be identified. Disadvantages of the technique include that it is less suitable for identifying hydrophobic substrates. The complex composition of body fluids may require. fractionation to limit the effect of regulators of the transporter which could mask transport of some substrates (Krumpochova et al., 2012).

After several years of tranquility, the expression profile of *ABCC6* has again been the subject of debate. ABCC6 is predominantly expressed at the basolateral side of liver and kidney cells, though the transporter also has differential expression in the gut and gastro-intestinal tract (Sinkó et al., 2003; Mutch, 2004; Pomozi et al., 2013). Recently, a supposed intracellular location in the mitochondria-associated membrane (MEM) – part of the ER complex – has been described (Martin et al., 2012). Though the shift in the paradigm linking the expression and function of ABCC6 to the hepatic and renal plasma membrane (PM), which is declared in this paper, should be reviewed with skepticism – the amount of evidence locating ABCC6 in the PM is after all overwhelming – the concept of an additional intracellular localization is potentially interesting and may further increase the complexity of the pathophysiological enigma at hand. This issue has been the topic of debate in recent papers by respectively Martin et al. (2012, 2013) and Pomozi et al. (2013). Demonstrating again the PM localization of ABCC6, the latter group could not confirm the MEM localization while the former called on methodological arguments to defend their findings (Martin et al., 2013; Pomozi et al., 2013). Challenging the cellular localization of proteins is not unprecedented in the ABC superfamily. Recently, the position of the ABCB6 transporter, originally thought to function in mitochondrial porphyrin metabolism, was challenged and extensively documented with experimental and literature data (Kiss et al., 2012). Despite the critical review and convincing evidence of the true physiological function of ABCB6, the authors did not completely exclude a contribution of ABCB6 to porphyrin metabolism and appealed for further and thorough study of the true pathophysiological function(s) of this transporter (Kiss et al., 2012). The knowledge on the subcellular localization of a native protein is of critical importance to model and understand the pathophysiology of any disease. Therefore, unity should be achieved regarding the PM localization of the ABCC6 transporter, but as with ABCB6 I would at this point not completely reject the idea of an additional intracellular localization of ABCC6. In view of the current contradictory results on the potential MEM localization, further work should be done to clarify this issue as the abnormal mitochondrial morphology and membrane potential in PXE – mentioned by Martin et al. (2012) as supporting evidence is insufficient; indeed, it has been demonstrated for the renal ABCB1 transporter that morphological abnormalities of the mitochondria can occur in the absence of a mitochondrial localization, possibly due to accumulation of toxic products (Huls et al., 2007). It cannot be excluded that ABCC6 may move between different membrane compartments under particular conditions, which theoretically may explain the contradicting findings for MEM localization. This has been shown for the PM ABCA1 transporter which, in the absence of extracellular Apo-AI, can be sequestered on intracellular membranes or degraded (Tang et al., 2009). As one particular protein can serve strictly different functions in separate parts of the same cell – in view of the complexity of what we know on PXE, this might not even be so unexpected – a validated intracellular localization of ABCC6 may help us further to understand the disease (Kumar and Snyder, 2002).

Though much attention has been paid to the expression of the ABCC6 transporter in the liver, its presence in the kidney, gut and the intestinal tract are less frequently the focus of attention. It has been shown for ABC transporters such as ABCC2 that the ABC protein may have different organ-specific functions (Jemnitz et al., 2010). Where in the liver it functions in biliairy transport, in the kidney it is involved in the excretion of small organic anions. A similar tissue-dependent function was shown for ABCA1 and ABCG1 (Tarling et al., 2013). Specifically addressing these extra-hepatic ABCC6 proteins is necessary as it is not unconceivable that for example the mucosal involvement in PXE may depend on or be modulated by the intestinal ABCC6 transporter.

## REGULATION OF ABCC6

Distinct pathways are involved in the regulation of ABC transporters, comprising genetic, epigenetic and nuclear-receptor mediated mechanisms, as well as post-transcriptional target repression by microRNAs (miRNAs), which can be triggered by hormones, growth factors and exogenous factors. Several examples exist of ABC transporters, such as Pgp, MRP4, BCRP, whose regulation depends on a combination of mechanisms (Masereeuw and Russel, 2012). Nuclear factor regulation and methylation dependent epigenetic regulation has been described for ABCC6 (Arányi et al., 2005; de Boussac et al., 2010; Ratajewski et al., 2012). So far, no correlation with the PXE phenotype, its variability or potential therapeutic approaches has been attempted. The influence of epigenetic changes, such as promotor DNA methylation, on disease variability has been shown for ABCA1 and coronary artery disease and various drug transporters such as ABCB1and MDR1 (Baker et al., 2005; Reed et al., 2008; Guay et al., 2012). Therapeutic consequences may include the use of histone deacytelase inhibitors, as reported for MDR1 (Jiang et al., 2009).

MicroRNA is a family of short non-coding RNAs involved in the negative regulation of gene expression at the posttranscriptional level (He and Hannon, 2004). With a few hundred miRNAs identified, it is estimated that 50% of the protein-coding genes are regulated by them. Among ABC transporters, miRNA dependent regulation has been documented for *CFTR*, *MRP2* and *4*, *PgP* and *BCRP*, either directly or indirectly through nuclear factors (Borel et al., 2012; Oglesby et al., 2013). In the *CFTR *gene, variants have been described that may influence the miRNA target sites in the 3′ UTR (Amato et al., 2013). It has been suggested that certain single nucleotide polymorphisms (SNPs) can influence the affinity for inhibitory miRNAs and may explain the differences in clinical expression between patients with an identical genotype. Mutations in the 3′ UTR have also been described in the *ABCC6* gene, so the involvement of miRNAs in PXE pathophysiology may not be purely theoretical. Identification of such miRNAs does not only open the possibility of using modulators, such as chemically engineered oligonucleotides called antagomirs, with the goal of influencing mechanisms that underlie disease initiation or progression (Masereeuw and Russel, 2012). Of interest is that miRNAs have been demonstrated useful biomarkers in kidney disease (Amato et al., 2013; De Guire et al., 2013). The development of an accurate biomarker set for PXE, which does not yet exist, will enable clinical studies to determine whether a compound has a clinically significant effect on the PXE phenotype.

## CLINICAL VARIABILITY

The clinical variability of the PXE phenotype remains a challenge for patients and physicians, making an individualized approach at this moment nearly impossible. Because of the lack of correlations between the patients phenotype and *ABCC6* genotype, the possibility of modifier genes has been suggested (Hendig et al., 2007; Zarbock et al., 2010, 2009). The number of potential modifier genes in PXE is currently still limited, which is not totally unexpected; for many ABC transporters the identification of clinical modulators remains challenging. The clinical variability in PXE shows similarities with the variability in cystic fibrosis (CF), the clinical course of which is also difficult to predict using the *ABCC7* mutations. Consequently, the quest for modifier genes has started and several modifiers of pulmonary outcome in CF have been described (Blaisdell et al., 2004; Boyle, 2007; Weiler and Drumm, 2012; von Kanel et al., 2013). The search for CF modifiers has lead to several recommendations which are equally valid for ABCC6. First, the importance of an in depth, unambiguous and universal definition of the phenotype has been deemed extremely important. Indeed, the description of the clinical features of the PXE phenotype is often inconsistent in different reports, even when tools such as the Phenodex^®^ are available (Pfendner et al., 2007). A more standardized definition of the phenotypic features will allow a more reliable identification and comparison of modifiers. Further, the limitations of association studies where the relationship between phenotype and polymorphisms in candidate modifier genes is examined have become clear, with the possibility of false positive studies or the causal effect of other genes which may travel with the candidate gene(s) (Nadeau, 2001; Accurso and Sontag, 2003). Finally, a study in twins and siblings in CF indicates that functions directly related to CFTR, membrane ion transport and/or intracellular trafficking of mutant protein are subject to modifier effects (Bronsveld et al., 2000, 2001). Identification of modifiers for functions directly related to the ABC transporter may also yield further insights in the pathophysiology of PXE and provide novel therapeutic targets. Next generation sequencing (NGS), a revolutionizing sequencing technique enabling parallel sequencing of multiple genes and whole exome sequencing is also an opportunity to identify modifier genes and variants, particularly in rare disorders in which large cohorts are often difficult to gather. By combining analysis of extreme phenotypes with pathway analysis, significant power can still be obtained, as was demonstrated by using NGS in CF (Emond et al., 2012).

Modifier genes may not be the only mechanism involved in the variability of PXE. For several ABC transporters, compensatory mechanisms have been described. These include upregulation of MRP3 expression in Dubin-Johnson syndrome, thus compensating for the impaired ABCC2 function (Masereeuw and Russel, 2012). Such compensatory mechanisms have also been suggested for PXE. Gene expression profiling of ABC transporters in dermal fibroblasts revealed increased expression of seven genes, including *ABCC2* and several members of the A-subfamily (Hendig et al., 2008). The latter was further explored in hepatocytes of a knock-out mouse model where tissue specific upregulation of *Abca4* was demonstrated in the liver (Li and Uitto, 2011). However, no further studies have investigated other potential compensatory mechanisms, though they may be of significant importance in understanding PXE, for aiming a more personalized follow-up of patients and for the introduction of novel therapeutic approaches.

## AN INTEGRATIVE APPROACH FOR PXE RESEARCH

Pseudoxanthoma elasticum is one of the diseases where, through the dedicated work of a relatively small group of researchers, a large number of data and observations are gathered. To take on the challenges that we are facing in the field of PXE, integration of all these data and findings may ultimately be the most difficult though imperative step to move forward efficiently. One interesting initiative in this respect is the Clinical and Functional Translation of CFTR (CFTR2) project, which presents a novel approach to clinical and functional annotation of mutations in the *CFTR* gene. Within this project, clinical and molecular data are gathered from CF registries and centers, in a standardized way and under the control of data managers. Further, data on functional assessment of mutations was added (Castellani and CFTR2 team, 2013). Though valuable initiatives have been taken to establish mutation database for *ABCC6*, and linking these molecular data to phenotypical characteristics, no such comprehensive database is currently available. A comprehensive database would improve our ability to identify biomarkers and interpret underlying mechanisms of disease variability in PXE. This database would ideally incorporate information on functional mutation data, potential or established modifier variants, exome sequencing data, serum measurements of patients, fibroblast observations, proteomics, metabolomics and other -omics in clinically well-characterized patients. This is expected to enhance diagnostics, carrier testing and screening, genotype-phenotype correlations, modifier analysis and insights into pathogenesis and therapies. It can be concluded that the other members of the ABC transporter family can provide us with valuable information and useful precedents for further characterizing the ABCC6 transporter. Perhaps the most important lesson to incorporate in current PXE research is the concept that only an integrative approach will finally enable us to elucidate this disease completely. To this purpose, it is therefore imperative that joint initiatives can be outlined to merge and integrate past, present and future research data.

## Conflict of Interest Statement

The authors declare that the research was conducted in the absence of any commercial or financial relationships that could be construed as a potential conflict of interest.

## References

[B1] Accurso F. J. Sontag M. K. (2003). Seeking modifier genes in cystic fibrosis. *Am. J. Respir. Crit. Care Med.* 167 289–290 10.1164/rccm.2210006 12554616

[B2] Amato F. Seia M. Giordano S. Elce A. Zarrilli F. Castaldo G. (2013). Gene mutation in microRNA target sites of CFTR gene: a novel pathogenetic mechanism in cystic fibrosis? *PLoS ONE* 8:e60448 10.1371/journal.pone.0060448 PMC3608608 23555973

[B3] Arányi T. Ratajewski M. Bardóczy V. Pulaski L. Bors A. Tordai A. (2005). Identification of a DNA methylation-dependent activator sequence in the pseudoxanthoma elasticum gene, ABCC6. *J. Biol. Chem.* 280 18643–18650 10.1074/jbc.M501139200 15760889

[B4] Baker E. K. Johnstone R. W. Zalcberg J. R. El-Osta A. (2005). Epigenetic changes to the MDR1 locus in response to chemotherapeutic drugs. *Oncogene* 24 8061–8075 10.1038/sj.onc.1208955 16091741

[B5] Bergen A. A. Plomp A. S. Schuurman E. J. Terry S. Breuning M. Dauwerse H. (2000). Mutations in ABCC6 cause pseudoxanthoma elasticum. *Nat. Genet.* 25 228–231 10.1038/76109 10835643

[B6] Blaisdell C. J. Howard T. D. Stern A. Bamford P. Bleecker E. R. Stine O. C. (2004). CLC-2 single nucleotide polymorphisms (SNPs) as potential modifiers of cystic fibrosis disease severity. *BMC Med. Genet. * 5:26 10.1186/1471-2350-5-26 PMC526769 15507145

[B7] Borel F. F. Han R. R. Visser A. A. Petry H. H. van Deventer S. J. Jansen P. L. (2012). Adenosine triphosphate-binding cassette transporter genes up-regulation in untreated hepatocellular carcinoma is mediated by cellular microRNAs. *Hepatology* 55 821–832 10.1002/hep.24682 21932399

[B8] Borst P. P. Zelcer N. N. van de Wetering K. K Poolman B. B. (2006). On the putative co-transport of drugs by multidrug resistance proteins. *FEBS Lett.* 580 9–9 10.1016/j.febslet.2005.12.039 16386247

[B9] Boyle M. P. (2007). Strategies for identifying modifier genes in cystic fibrosis. *Proc. Am. Thorac. Soc.* 4 52–57 10.1513/pats.200605-129JG 17202292 PMC2647615

[B10] Bronsveld I. Mekus F. Bijman J. Ballmann M. de Jonge H. R. Laabs U. (2001). Chloride conductance and genetic background modulate the cystic fibrosis phenotype of Delta F508 homozygous twins and siblings. *J. Clin. Invest.* 108 1705–1715 10.1172/JCI12108 11733566 PMC200980

[B11] Bronsveld I. Mekus F. Bijman J. Ballmann M. Greipel J. Hundrieser J. (2000). Residual chloride secretion in intestinal tissue of deltaF508 homozygous twins and siblings with cystic fibrosis. The European CF Twin and Sibling Study Consortium. *Gastroenterology* 119 32–40 10.1053/gast.2000.8524 10889152

[B12] Castellani C CFTR2 team. (2013). CFTR2: how will it help care? *Paediatr. Respir. Rev.* 14(Suppl. 1) 2–5 10.1016/j.prrv.2013.01.006 23466340

[B13] Chassaing N. Martin L. Calvas P. Le Bert M. Hovnanian A. (2005). Pseudoxanthoma elasticum: a clinical, pathophysiological and genetic update including 11 novel ABCC6 mutations. *J. Med. Genet.* 42 881–892 10.1136/jmg.2004.030171 15894595 PMC1735972

[B14] Ci L. Kusuhara H. Adachi M. Schuetz J. D. Takeuchi K. Sugiyama Y. (2007). Involvement of MRP4 (ABCC4) in the luminal efflux of ceftizoxime and cefazolin in the kidney. *Mol. Pharmacol.* 71 1591–1597 10.1124/mol.106.031823 17344354

[B15] de Boussac H. Ratajewski M. Sachrajda I. Köblös G. Tordai A. Pulaski L. (2010). The ERK1/2-hepatocyte nuclear factor 4alpha axis regulates human ABCC6 gene expression in hepatocytes. *J. Biol. Chem.* 285 22800–22808 10.1074/jbc.M110.105593 20463007 PMC2906271

[B16] De Guire V. Robitaille R. Tétreault N. Guérin R. Ménard C. Bambace N. (2013). Circulating miRNAs as sensitive and specific biomarkers for the diagnosis and monitoring of human diseases: promises and challenges. *Clin. Biochem.* 46 846–860 10.1016/j.clinbiochem.2013.03.015 23562576

[B17] Emond M. J. Louie T. Emerson J. Zhao W. Mathias R. A. Knowles M. R. (2012). Exome sequencing of extreme phenotypes identifies DCTN4 as a modifier of chronic *Pseudomonas aeruginosa* infection in cystic fibrosis. *Nat. Genet.* 44 886–889 10.1038/ng.2344 22772370 PMC3702264

[B18] Ferreira R. J. Ferreira M. J Santos Dos D. J. (2013). Molecular docking characterizes substrate-binding sites and efflux modulation mechanisms within P-glycoprotein. *J. Chem. Inf. Model.* 53 1747–6170 10.1021/ci400195v 23802684

[B19] Gheduzzi D. Guidetti R. Anzivino C. Tarugi P. Di Leo E. Quaglino D. (2004). ABCC6 mutations in Italian families affected by pseudoxanthoma elasticum (PXE). *Hum. Mutat.* 24 438–439 10.1002/humu.9284 15459974

[B20] Glavinas H. Krajcsi P. Cserepes J. Sarkadi B. (2004). The role of ABC transporters in drug resistance, metabolism and toxicity. *Curr. Drug Deliv.* 1 27–42 10.2174/1567201043480036 16305368

[B21] Guay S.-P. S. Brisson D. D. Munger J. J. Lamarche B. B. Gaudet D. D. Bouchard L. L. (2012). ABCA1 gene promoter DNA methylation is associated with HDL particle profile and coronary artery disease in familial hypercholesterolemia. *Epigenetics* 7 464–472 10.4161/epi.19633 22419126

[B22] He L. Hannon G. J. (2004). MicroRNAs: small RNAs with a big role in gene regulation. *Nat. Rev. Genet.* 5 522–531 10.1038/nrg1379 15211354

[B23] He S.-M. Li R. Kanwar J. R. Zhou S.-F. (2011). Structural and functional properties of human multidrug resistance protein 1 (MRP1/ABCC1). *Curr. Med. Chem.* 18 439–481 10.2174/092986711794839197 21143116

[B24] Hendig D. Arndt M. Szliska C. Kleesiek K Götting C. (2007). SPP1 promoter polymorphisms: identification of the first modifier gene for pseudoxanthoma elasticum. *Clin. Chem.* 53 829–836 10.1373/clinchem.2006.083675 17384004

[B25] Hendig D. Langmann T. Kocken S. Zarbock R. Szliska C. Schmitz G. (2008). Gene expression profiling of ABC transporters in dermal fibroblasts of pseudoxanthoma elasticum patients identifies new candidates involved in PXE pathogenesis. *Lab. Invest.* 88 1303–1315 10.1038/labinvest.2008.96 18936737

[B26] Hirono S. S. Nakagome I. I. Imai R. R. Maeda K. K. Kusuhara H. H. Sugiyama Y. Y. (2005). Estimation of the three-dimensional pharmacophore of ligands for rat multidrug-resistance-associated protein 2 using ligand-based drug design techniques. *Pharm. Res.* 22 260–269 10.1007/s01869-005-1869-8 15783074

[B27] Huls M. Kramers C. Levtchenko E. N. Wilmer M. J. Dijkman H. B. Kluijtmans L. A. (2007). P-glycoprotein-deficient mice have proximal tubule dysfunction but are protected against ischemic renal injury. *Kidney Int.* 72 1233–1241 10.1038/sj.ki.5002522 17851469

[B28] Ivan S. Breljak D. Marija L. Hrvoje B. (2013). Are mice, rats, and rabbits good models for physiological, pharmacological and toxicological studies in humans? *Period. Biol.* 113 7–26

[B29] Jemnitz K. K. Heredi-Szabo K. K. Janossy J. J. Ioja E. E. Vereczkey L. L. Krajcsi P. P. (2010). ABCC2/Abcc2: a multispecific transporter with dominant excretory functions. *Drug Metab. Rev.* 42 402–436 10.3109/03602530903491741 20082599

[B30] Jiang Z.-P. Xu P. Wang G.-P. Zhao X.-L. Chen F.-P. (2009). Hypothesizing that histone deacetylase inhibitors can be used to reverse multiple drug resistance. *Med. Hypotheses* 74 3–3 10.1016/j.mehy.2009.07.036 19700246

[B31] Katona M. Kiss K. Angyal V. Kucsma N. Sarkadi B. Takáts Z. (2009). A mass spectrometry based functional assay for the quantitative assessment of ABC transporter activity. *Rapid Commun. Mass Spectrom.* 23 3372–3376 10.1002/rcm.4259 19780062

[B32] Kiss K. Brozik A. Kucsma N. Toth A. Gera M. Berry L. (2012). Shifting the paradigm: the putative mitochondrial protein ABCB6 resides in the lysosomes of cells and in the plasma membrane of erythrocytes. *PLoS ONE * 7:e37378 10.1371/journal.pone.0037378 PMC3360040 22655043

[B33] Krumpochova P. P. Sapthu S. S. Brouwers J. F. J. de Haas M. M. de Vos R. R. Borst P. P. (2012). Transportomics: screening for substrates of ABC transporters in body fluids using vesicular transport assays. *FASEB J.* 26 738–747 10.1096/fj.11-195743 22034653

[B34] Kumar A. A. Snyder M. M. (2002). Protein complexes take the bait. *Nature* 415 123–124 10.1038/415123a 11805813

[B35] Le Saux O. Bunda S. VanWart C. M. Douet V. Got L. Martin L. (2006). Serum factors from pseudoxanthoma elasticum patients alter elastic fiber formation *in vitro*. *J. Invest. Dermatol.* 126 1497–1505 10.1038/sj.jid.5700201 16543900 PMC5540375

[B36] Le Saux O. Urban Z. Tschuch C. Csiszar K. Bacchelli B. Quaglino D. (2000). Mutations in a gene encoding an ABC transporter cause pseudoxanthoma elasticum. *Nat. Genet.* 25 223–227 10.1038/76102 10835642

[B37] Li Q. Uitto J. (2011). Expression of the Abca-subfamily of genes in Abcc6-/- mice–upregulation of Abca4. *Exp. Dermatol.* 20 452–454 10.1111/j.1600-0625.2010.01240.x 21435020 PMC3079327

[B38] Luckie D. Wilterding J. Krha M. Krouse M. (2003). CFTR and MDR: ABC transporters with homologous structure but divergent function. *Curr. Genomics* 4 225–235 10.2174/1389202033490394

[B39] Madon J. Hagenbuch B. Landmann L. Meier P. J. Stieger B. (2000). Transport function and hepatocellular localization of mrp6 in rat liver. *Mol. Pharmacol.* 57 634–641 10692506 10.1124/mol.57.3.634

[B40] Martin L. J. Lau E. Singh H. Vergnes L. Tarling E. J. Mehrabian M. (2012). ABCC6 localizes to the mitochondria-associated membrane. *Circ. Res.* 111 516–520 10.1161/CIRCRESAHA.112.276667 22811557 PMC3540978

[B41] Martin L. Lau E. Singh H. Vergnes L. Tarling E. Mehrabian M. (2013). Response to Pomozi et al’s research commentary. *Circ. Res.* 112 e152–e153 10.1161/CIRCRESAHA.113.301666 23625949 PMC3999040

[B42] Masereeuw R Russel F. G. M. (2012). Regulatory pathways for ATP-binding cassette transport proteins in kidney proximal tubules. *AAPS J.* 14 883–894 10.1208/s12248-012-9404-z 22961390 PMC3475864

[B43] Meloni I. Rubegni P. De Aloe G. Bruttini M. Pianigiani E. Cusano R. (2001). Pseudoxanthoma elasticum: point mutations in the ABCC6 gene and a large deletion including also ABCC1 and MYH11. *Hum. Mutat.* 18 85 10.1002/humu.1157 11439001

[B44] Mutch D. M. (2004). Regional variations in ABC transporter expression along the mouse intestinal tract. *Physiol. Genomics* 17 11–20 10.1152/physiolgenomics.00150.2003 14679303

[B45] Nadeau J. H. (2001). Modifier genes in mice and humans. *Nat. Rev. Genet.* 2 165–174 10.1038/35056009 11256068

[B46] Nakayama A. Matsuo H. Takada T. Ichida K. Nakamura T. Ikebuchi Y. (2011). ABCG2 is a High-capacity urate transporter and its genetic impairment increases serum uric acid levels in humans. *Nucleosides Nucleotides Nucleic Acids* 30 1091–1097 10.1080/15257770.2011.633953 22132962

[B47] Nitschke Y. Baujat G. Botschen U. Wittkampf T. Moulin, du M. Stella J. (2011). Generalized arterial calcification of infancy and pseudoxanthoma eElasticum can be caused by mutations in either ENPP1 or ABCC6. *Am. J. Hum. Genet.* 90 25–39 10.1016/j.ajhg.2011.11.020 22209248 PMC3257960

[B48] Oglesby I. K. Chotirmall S. H. McElvaney N. G. Greene C. M. (2013). Regulation of cystic fibrosis transmembrane conductance regulator by microRNA-145, -223, and -494 is altered in ΔF508 cystic fibrosis airway epithelium. *J. Immunol.* 190 3354–3362 10.4049/jimmunol.1202960 23436935

[B49] Pasquali-Ronchetti I. Garcia-Fernandez M. I. Boraldi F. Quaglino D. Gheduzzi D. De Vincenzi Paolinelli C. (2006). Oxidative stress in fibroblasts from patients with pseudoxanthoma elasticum: possible role in the pathogenesis of clinical manifestations. *J. Pathol.* 208 54–61 10.1002/path.1867 16261549

[B50] Pedersen J. M. Matsson P. Bergström C. A. S. Norinder U. Hoogstraate J. Artursson P. (2008). Prediction and identification of drug interactions with the human ATP-binding cassette transporter multidrug-resistance associated protein 2 (MRP2; ABCC2). *J. Med. Chem.* 51 3275–3287 10.1021/jm7015683 18457386

[B51] Pfendner E. G. Vanakker O. M. Terry S. F. Vourthis S. McAndrew P. E. McClain M. R. (2007). Mutation detection in the ABCC6 gene and genotype-phenotype analysis in a large international case series affected by pseudoxanthoma elasticum. *J. Med. Genet.* 44 621–628 10.1136/jmg.2007.051094 17617515 PMC2597973

[B52] Pomozi V. Le Saux O. Brampton C. Apana A. Iliás A. Szeri F. (2013). ABCC6 is a basolateral plasma membrane protein. *Circ. Res.* 112 e148–e151 10.1161/CIRCRESAHA.111.300194 23625951 PMC3727999

[B53] Ratajewski M. de Boussac H. Sachrajda I. Váradi A Arányi T. (2012). ABCC6 expression is regulated by CCAAT/enhancer binding protein activating a primate-specific sequence located in the first intron of the gene. *J. Invest. Dermatol.* 132 2709–2717 10.1038/jid.2012.218 22763786 PMC3509247

[B54] Reed K. Hembruff S. L. Laberge M. L. Villeneuve D. J. Côté G. B. Parissenti A. M. (2008). Hypermethylation of the ABCB1 downstream gene promoter accompanies ABCB1 gene amplification and increased expression in docetaxel-resistant MCF-7 breast tumor cells. *Epigenetics* 3 270–280 10.4161/epi.3.5.6868 19001875

[B55] Sinkó E. Iliás A. Ujhelly O. Homolya L. Scheffer G. L. Bergen A. A. B. (2003). Subcellular localization and N-glycosylation of human ABCC6, expressed in MDCKII cells. *Biochem. Biophys. Res. Commun.* 308 263–269 10.1016/S0006-291X(03)01349-4 12901863

[B56] Tang C. C. Liu Y. Y. Kessler P. S. P. Vaughan A. M. A Oram J. F. J. (2009). The macrophage cholesterol exporter ABCA1 functions as an anti-inflammatory receptor. *J. Biol. Chem.* 284 32336–32343 10.1074/jbc.M109.047472 19783654 PMC2781648

[B57] Tarling E. J. Vallim T. Q. de A. Edwards P. A. (2013). Role of ABC transporters in lipid transport and human disease. *Trends Endocrinol. Metab.* 24 342–350 10.1016/j.tem.2013.01.006 23415156 PMC3659191

[B58] Vanakker O. M. Leroy B. P. Coucke P. Bercovitch L. G. Uitto J. Viljoen D. (2008). Novel clinico-molecular insights in pseudoxanthoma elasticum provide an efficient molecular screening method and a comprehensive diagnostic flowchart. *Hum. Mutat.* 29 205 10.1002/humu.9514 18157818

[B59] Vanakker O. M. Martin L. Schurgers L. J. Quaglino D. Costrop L. Vermeer C. (2010). Low serum vitamin K in PXE results in defective carboxylation of mineralization inhibitors similar to the GGCX mutations in the PXE-like syndrome. *Lab. Invest.* 90 895–905 10.1038/labinvest.2010.68 20368697

[B60] von Kanel T. Stanke F. Weber M. Schaller A. Racine J. Kraemer R. (2013). Clinical and molecular characterization of the potential CF disease modifier syntaxin 1A. *Eur. J. Hum. Genet.* 10.1038/ejhg.2013.57 [Epub ahead of print] PMC3831073 23572023

[B61] Weiler C. A. Drumm M. L. (2012). Genetic influences on cystic fibrosis lung disease severity. *Front. Pharmacol. * 4:40–40 10.3389/fphar.2013.00040 23630497 PMC3632778

[B62] Zarbock R. Hendig D. Szliska C. Kleesiek K. Gotting C. (2009). Vascular endothelial growth factor gene polymorphisms as prognostic markers for ocular manifestations in pseudoxanthoma elasticum. *Hum. Mol. Genet.* 18 3344–3351 10.1093/hmg/ddp259 19483196

[B63] Zarbock R. Hendig D. Szliska C. Kleesiek K Götting C. (2010). Analysis of MMP2 promoter polymorphisms in patients with pseudoxanthoma elasticum. *Clin. Chim. Acta* 411 1487–1490 10.1016/j.cca.2010.06.006 20541540

